# Factors associated with antidepressant, anxiolytic and hypnotic use over 17 years in a national cohort

**DOI:** 10.1016/j.jad.2008.01.021

**Published:** 2008-10

**Authors:** Ian Colman, Tim J. Croudace, Michael E.J. Wadsworth, Peter B. Jones

**Affiliations:** a Department of Public Health Sciences, School of Public Health, University of Alberta, Edmonton, Canada; b Department of Psychiatry, University of Cambridge, Cambridge, United Kingdom; c Medical Research Council National Survey of Health and Development, Department of Epidemiology and Public Health, University College London Medical School, London, United Kingdom

**Keywords:** Antidepressants, Anxiolytics, Hypnotics, Treatment, Depression, Anxiety

## Abstract

**Background:**

In the general population, most individuals with mental disorders are not treated with psychotropic medications. The objective of this study was to identify factors associated with psychotropic medication use over a 17 year period in a birth cohort.

**Method:**

Members of the 1946 British birth cohort (*n* = 2928 in 1999) reported psychotropic medication use in 1982 at age 36, in 1989 at age 43, and in 1999 at age 53. At each of the three time points, several factors were investigated for their association with antidepressant, anxiolytic or hypnotic medication use.

**Results:**

After adjusting for severity of symptoms of depression and anxiety, clinical factors such as suicidal ideation, sleep difficulty and poor physical health were strongly associated with antidepressant, anxiolytic or hypnotic medication use in 1982 and 1989, but not in 1999. Non-clinical factors were infrequently associated with antidepressant, anxiolytic or hypnotic medication use in 1982 and 1989 after adjusting for severity of symptoms, however several non-clinical factors were associated with antidepressant, anxiolytic or hypnotic medication use in 1999 including being female (OR = 1.4, 95% CI: 1.0, 1.9), unemployment (OR = 2.9, 95% CI: 2.1, 4.1), living alone (OR = 2.6, 95% CI: 1.7, 3.9), and being divorced, separated or widowed (OR = 1.5, 95% CI: 1.1, 2.3).

**Limitations:**

Data were not available on help-seeking behaviour.

**Conclusions:**

Treatment of mental disorder with psychotropic medications is strongly associated with clinical factors. However, non-clinical factors continue to be significant, and may influence both treatment-seeking and prescribing behaviour.

## 1 Introduction

Epidemiological studies in the U.S. and Europe (Burns et al., 2003; Colman et al., 2006; Linden et al., 1999; Simon et al., 2004; Spijker et al., 2001) have shown that the majority of individuals suffering from depression and similar common mental disorders do not seek or receive any treatment; reported rates of treatment with psychotropic medications range from 23 to 43% (Colman et al., 2006; Linden et al., 1999; Spijker et al., 2001). The 1997 U.S. National Depressive and Manic-Depressive Association Consensus Statement on the Undertreatment of Depression concluded that a pertinent area of research is identifying patient-related factors that contribute to undertreatment (Hirschfeld et al., 1997).

Clinical characteristics such as severity of current symptoms and a history of mental illness have been consistently linked to help-seeking behaviour and treatment with psychotropic medications among those with depression. For example, severity of symptoms (Andrews et al., 2001; Bebbington et al., 2000; Burns et al., 2003; Linden et al., 1999; Mojtabai et al., 2002; Simon et al., 2004; Spijker et al., 2001), suicidal ideation (Burns et al., 2003; Mojtabai et al., 2002), duration of illness (Blumenthal and Endicott, 1996; Spijker et al., 2001), and a history of psychiatric treatment (Blumenthal and Endicott, 1996; Burns et al., 2003) have been linked to treatment for mental illness. However, non-clinical factors have also been shown to predict treatment, and may be more strongly associated with treatment than clinical factors (Linden et al., 1999; Simon et al., 2004).

For example, many studies have shown that women (Andrews et al., 2001; Bebbington et al., 2000; Linden et al., 1999; Mojtabai et al., 2002) and those who are divorced, separated or widowed (Andrews et al., 2001; Bebbington et al., 2000; Linden et al., 1999; Mojtabai et al., 2002), unemployed (Bebbington et al., 2000; Linden et al., 1999; Spijker et al., 2001) or highly educated (Andrews et al., 2001; Blumenthal and Endicott, 1996) are all more likely to seek and receive treatment for common mental disorders.

To date, little research has examined whether the predictive nature of these factors has changed over time. The objective of this study was to identify factors associated with antidepressant, anxiolytic or hypnotic use during three assessments over a 17-year period, and to describe changes in the predictive nature of these factors over time. We hypothesized that, given efforts to educate the British population about depression such as the “Defeat Depression Campaign” (Paykel et al., 1998) and the proliferation of clinical guidelines that might standardize care such as the National Institute for Clinical Excellence Clinical Guideline for Management of Depression in Primary and Secondary Care (National Institute for Clinical Excellence, 2004), non-clinical factors such as sociodemographics, personality and socio-economic characteristics would be less important in more recent assessments, after adjusting for severity of psychiatric symptoms.

## 2 Method

### 2.1 Subjects

Subjects were members of the Medical Research Council National Survey of Health and Development (NSHD). This is a longitudinal study of 5362 individuals born in England, Scotland, or Wales, during the week of March 3–9, 1946. The sample has been prospectively studied on 21 occasions up to age 53. Comparisons with census data show that those remaining in the cohort (*n* = 3673 at age 53) are broadly representative of all native-born adults currently resident in Great Britain (Wadsworth et al., 2003).

This paper reports on data collected at three occasions: in 1982 at age 36 (*n* = 3248), in 1989 at age 43 (*n* = 3197), and in 1999 at age 53 (*n* = 2928).

### 2.2 Antidepressant, anxiolytic and hypnotic use

Survey members reported prescription medication use to a nurse interviewer in 1982, 1989 and 1999. Medications were then matched to a British National Formulary Volume 44 (BNF) code by a trained research nurse. Medications described in this report are: hypnotics (Section 4.1.1 of the BNF), anxiolytics (Section 4.1.2 of the BNF), and antidepressants (Section 4.3 of the BNF). As the focus of our analysis was treatment of common psychiatric symptoms, we excluded individuals who reported use of antipsychotics (Sections 4.2.1 and 4.2.2 of the BNF) or mood stabilizers (Section 4.2.3 of the BNF) in 1982, 1989 or 1999 (*n* = 52).

### 2.3 Predictors of antidepressant, anxiolytic and hypnotic use

Numerous factors were considered for possible associations with treatment with psychotropic medications. First was the presence of mental disorder. Additional predictors can be broadly grouped into clinical and non-clinical characteristics. Among the clinical characteristics, both current clinical features and psychiatric history were considered. Among non-clinical characteristics, personality, social and related factors, and factors describing the survey member's parents and childhood were considered.

#### 2.3.1 Mental disorder

Survey members were considered to have mental disorder in 1982 if they scored 5 or higher on the Index of Definition of the Present State Examination (PSE; Wing et al., 1978), yielding a prevalence of 5.9%. This prevalence was used to define mental disorder at ages 43 and 53 (Colman et al., 2006). Consequently, survey members were considered to have mental disorder in 1989 if they scored 31 or higher on the Psychiatric Symptom Frequency scale (PSF; Lindelow et al., 1997), and were considered to have mental disorder in 1999 if they scored 12 or higher on 28-item General Health Questionnaire (GHQ; Goldberg and Hillier, 1979).

#### 2.3.2 Current clinical features

Three current clinical characteristics were considered: suicidal ideation, sleep difficulty, and poor physical health. Survey members were considered to have suicidal ideation in 1982 if they reported during the PSE they had “deliberately considered suicide”, in 1989 if they reported during the PSF they had “thought about taking their own life”, and in 1999 if they “definitely thought about making away with themselves” or “definitely found that the idea of taking their life kept coming into their minds”. Survey members were considered to have trouble sleeping if they answered positively to any question regarding sleep difficulties on the PSE or GHQ in 1982 and 1999, respectively, and if they reported trouble sleeping in 1989. Finally, survey members were considered to be in poor physical health if they did not report feeling “physically very fit” on the PSE or GHQ in 1982 and 1999, respectively.

#### 2.3.3 Psychiatric history

Four variables were used to assess the psychiatric history: adolescent mental disorder, psychiatric treatment between age 16 and 36, self-reported history of nervous trouble and previous psychotropic medication use.

Survey members were assessed to have adolescent mental disorder based on based on questionnaires completed by teachers when the survey members were 13 and 15 years of age. These questionnaires have previously been subjected to factor analysis, with one factor being identified as anxious and depressive symptoms (Colman et al., 2007).

NSHD staff has previously used in- and out-patient hospital records and contact with physicians to assess whether a survey member had psychiatric treatment between the ages of 16 and 36 (Paykel et al., 2006).

Survey members reported if they had ever suffered from “nervous trouble” in 1982 and 1989. Survey members were considered to have previous psychotropic medication use if they had reported psychotropic medication use in a postal questionnaire in 1977, or used psychotropic medications in 1982 or 1989.

#### 2.3.4 Personality

Neurotic and extravert personality traits were measured using the Maudsley Personality Inventory at age 16 (Eysenck, 1958). Individuals were considered to have high neuroticism or extraversion if they scored 7 or higher on a scale from 0 to 12.

#### 2.3.5 Social and related factors

Survey members were asked to report on a number of general sociodemographic factors in 1982, 1989 and 1999, including their employment status, their marital status, and whether they lived alone. Social class was based on the British Registrar General's social class classification according to the current or last occupation of the survey member (Kuh et al., 2005); members were grouped into manual vs. non-manual social classes. Highest level of education was measured based on examination reports and by self-report at age 26. Survey members were grouped into those with ‘ordinary’ qualifications (O-levels or lower) or ‘advanced’ qualifications (A-levels or higher) (Richards et al., 2001).

In 1982, survey members reported the occurrence of eight stressful life events (SLEs) in the previous 12 months, while in 1989 and 1999 the occurrence of 17 SLEs in the previous 12 months were recorded (van Os et al., 2001). Survey members were grouped into those who had one or no SLEs in the previous year versus those with more. In addition, the sex of the survey member was included in this section.

#### 2.3.6 Parental and childhood factors

In 1989, survey members were asked if either of their parents had trouble with their nerves. This retrospective measure was used in all three sub-samples.

Survey members' cognitive ability was measured at age 8 using reading comprehension, word pronunciation, vocabulary, and non-verbal reasoning tests. These test scores have been combined to create a global cognitive ability (Richards et al., 2001). Survey members were grouped according to whether they were above (i.e., high cognitive ability) or below the median on this composite score. In addition, the social class of the father as described above was included in this section.

### 2.4 Statistical analysis

The predictors listed above were tested for their association with drug treatment in 1982, 1989 and 1999 using logistic regression. All predictive factors, apart from mental disorder, were tested for their association with drug treatment after controlling for severity of psychiatric symptoms using the continuous scores (i.e., Index of Definition for the PSE at age 36, PSF score at age 43, and GHQ score at age 53). Associations are presented as odds ratios (ORs) with 95% confidence intervals (95% CI).

## 3 Results

In 1982, at age 36, 145 individuals (4.5%) were using an antidepressant, anxiolytic or hypnotic. In 1989, at age 43, 144 individuals (4.5%) were using one of these psychotropic medications. In 1999, at age 53, 174 individuals (5.9%) were using an antidepressant, anxiolytic or hypnotic.

### 3.1 Clinical predictors of antidepressant, anxiolytic or hypnotic use

The presence of mental disorder was strongly associated with the likelihood of using an antidepressant, anxiolytic or hypnotic in 1982, 1989 and 1999 (see Table 1). In addition, suicidal ideation, sleep difficulties, and poor physical health were all associated with drug treatment after adjusting for severity of symptoms in 1982 and 1989, but not in 1999.

A history of psychiatric symptoms or treatment was also strongly associated with the likelihood of being treated with an antidepressant, anxiolytic or hypnotic (see Table 1). Previous psychotropic medication use was a consistent predictor of current use. Similarly, psychiatric treatment during early adulthood was associated with psychotropic medication use. In addition, a self-reported history of nervous trouble was also associated with medication use.

### 3.2 Non-clinical predictors of antidepressant, anxiolytic or hypnotic use

Non-clinical characteristics were less consistently associated with antidepressant, anxiolytic or hypnotic use, after controlling for severity of psychiatric symptoms (see Table 2). In 1982, unemployed and less neurotic individuals were more likely to be using psychotropic medications. In 1989, females and those whose parents had nervous trouble were more likely to be using psychotropic medications. Several non-clinical factors were associated with antidepressant, anxiolytic or hypnotic use in 1999. Females and those whose parents had nervous trouble were again more likely to be using psychotropic medications, as were those who were neurotic, living alone, unemployed, and divorced, separated or widowed.

## 4 Discussion

### 4.1 The findings and existing research

Contrary to our original hypothesis, clinical predictors were less likely to be associated with the use of antidepressants, anxiolytics or hypnotics in 1999 as compared with 1982 or 1989. For example, suicidal ideation, difficulty sleeping, and poor physical health were associated with psychotropic medication use in 1982 and 1989, but none of these factors were associated with psychotropic drug use in 1999. On the other hand, non-clinical factors were more likely to be associated with antidepressant, anxiolytic or hypnotic use in 1999 than in 1982 or 1989, after adjusting for severity of psychiatric symptoms.

One of the factors very strongly associated with antidepressant, anxiolytic and hypnotic use in this study was the individual reporting a history of nervous trouble. An important aspect of this variable is that individuals who report a history of nervous trouble are acknowledging that they have had problems with mental health. Those with mental health difficulties are not always likely to acknowledge current or present symptoms; among NSHD survey members who had mental disorder in adolescence, the majority do not report a history of nervous trouble as little as ten years later (Colman et al., 2007). One of the key barriers to treatment of depression is whether an individual recognises the fact that they have an illness (Docherty, 1997). This is related to the concept of perceived need for treatment. Psychological models of perceived need identify three stages that an individual goes through before perceiving a need for treatment: experiencing symptoms, evaluating the significance of these symptoms and possible consequences, and determining whether treatment may be beneficial (Mojtabai et al., 2002). Perceived need for care is strongly associated with help-seeking behaviour for those with symptoms of mental illness (Andrews et al., 2001; Burns et al., 2003; Mojtabai et al., 2002).

Another strong predictor of antidepressant, anxiolytic and hypnotic use was having employed similar strategies to deal with mental illness in the past. These variables comprise two factors: a past psychiatric history, and a demonstrated willingness to seek and receive treatment. Having a positive attitude towards mental health treatment is associated with help-seeking behaviour (Mojtabai et al., 2002); if patients have demonstrated a previous positive attitude towards mental health treatment then they are likely to use future treatment. However, among individuals who recognise that they could benefit from help with their mental health, many do not seek or choose to accept any form of treatment (Andrews et al., 2001; Mojtabai et al., 2002). Many prefer to manage on their own (Andrews et al., 2001; Blumenthal and Endicott, 1996; Burns et al., 2003), or depend upon friends or family for help (Burns et al., 2003). This may be because individuals are embarrassed or afraid to speak to a health professional about their mental health (Blumenthal and Endicott, 1996; Simon et al., 2004), or fear possible consequences in their career or personal relationships (Blumenthal and Endicott, 1996; Simon et al., 2004).

It is notable that non-clinical factors like unemployment and lack of social support were associated with antidepressant, anxiolytic and hypnotic use in 1999, which may reflect physician behaviour or attitudes to their patient's social and economic situations. This may be particularly relevant when social or occupational influences have been present for some time (e.g., unemployment), and thought to influence the persistence of the episode or prognosis of the illness. Since many cases of depression remit spontaneously regardless of treatment (Simon et al., 1999), physicians may choose to not treat a proportion of patients, expecting the symptoms to subside naturally. A recent British study reported that physicians' preferred strategy for dealing with depression in primary care is to ‘wait and see’ if symptoms remit without treatment (Hyde et al., 2005). This strategy is most likely in cases where symptoms are milder and of lesser duration. However, physicians may be less likely to delay treatment in certain contexts where consequences of non-treatment may be more severe such as unemployment.

It is also notable that females were more likely to be using antidepressants, anxiolytics or hypnotics in 1989 and 1999. It has been shown that females are more likely to perceive a need for help when they have symptoms of mental disorder (Mojtabai et al., 2002), and consequently they are more likely to seek treatment for these symptoms (Andrews et al., 2001; Bebbington et al., 2000). Similarly, those whose parents had trouble with nerves were more likely to be using antidepressants, anxiolytics or hypnotics in 1989 and 1999. It has been suggested that these individuals may be more likely to recognize symptoms of mental illness and may be more likely to then seek help (Mojtabai et al., 2002). Individuals whose parents have a history of mental illness may also have witnessed successful psychiatric treatment for their parents, which could positively influence their decision to seek help.

The fact that the factors most strongly associated with antidepressant, anxiolytic and hypnotic use were clinical ones is a positive finding. It is, however, somewhat disconcerting that there were several non-clinical factors associated with psychotropic medication use in 1999, particularly if these were influenced by physician behaviour. As one commentator has observed, “The appropriateness of current psychotropic drug prescribing practices has been repeatedly questioned. The importance of non-clinical factors in drug prescribing is one major source of concern.” (Linden et al., 1999) To that end, we support those who have called for better education of general practitioners in how to deal with patients with psychiatric symptoms (Andrews et al., 2001; Bebbington et al., 2000; Linden et al., 1999). A notable study in Sweden reported that an education program supporting comprehensive care for depression by general practitioners significantly reduced rates of in-patient treatment of depression and significantly reduced rates of suicide (Rutz, 2001).

With regards to treatment-seeking behaviour, many have noted that the majority of those with mental disorder do not seek treatment for their symptoms (Andrews et al., 2001; Bebbington et al., 2000; Blumenthal and Endicott, 1996; Colman et al., 2006). To help improve this number, we support efforts such as the Defeat Depression Campaign (Paykel et al., 1998) and those who call for further attempts to educate the public about mental illness and appropriate treatment (Andrews et al., 2001; Bebbington et al., 2000).

### 4.2 Methodological considerations

A notable limitation of this study is that the outcome, treatment with psychotropic medications, is influenced by two key players: patients, who must recognize their symptoms and decide whether to seek treatment, and physicians, who must diagnose a mental illness decide whether or not to prescribe psychotropic drugs. It is possible that factors that influence patient behaviour are different from those that influence physician behaviour. Data was available in this cohort on treatment-seeking behaviour in this cohort in 1982 and 1989 in the form of the question “have you visited a physician in the past year for treatment for nerves” (data not shown). Analysis of these data indicated that there were few differences between predictors of treatment-seeking behaviour and predictors among drug treatment among those who sought treatment.

The second limitation is that it is not possible to separate age and period effects in this cohort study. While we conclude that observed changes in the predictive nature of clinical and non-clinical factors with regards to antidepressant, anxiolytic and hypnotic use are a result of period effects over a seventeen-year period, it may be that these changes are a result of shifts in physician and patient behaviour as individuals age from early- to mid-adulthood. We believe this is unlikely, given that the time period from age 36 to 53 is not a time of great change relative to other stages of the life course, but it is possible.

These limitations are offset by some notable strengths of the study. First, we used data collected at three different time points over a 17-year period. Second, we had a large sample size at each collection point that was drawn from a population-based cohort. Finally, we report on a large number of individual factors that have been posited to be associated with psychotropic medication use.

### 4.3 Conclusion

Treatment of mental disorder with psychotropic medications at the population level is strongly predicted by clinical factors. However non-clinical factors continue to be important and may influence both treatment-seeking and prescribing behaviour. The results support improved education concerning treatment of common mental disorders for both physicians and the public.

## Role of funding source

The funding bodies had no involvement with the design and analysis of the study, or the interpretation and presentation of the results.

## Conflicts of interest

We have no conflicts of interest to declare.

## Figures and Tables

**Table 1:** Clinical predictors of those treated with psychotropic medications during an episode of mental disorder

	Odd ratios for treatment (95% CI)^a^		
	1982	1989	1999
	Age 36	Age 43	Age 53
	*n* = 3248	*n* = 3197	*n* = 2928
Mental disorder	**9.1 (6.2, 13.4)**	**11.9 (8.1, 17.3)**	**7.8 (5.4, 11.4)**
			
*Current clinical symptoms*			
Current suicidal ideation	**3.1 (1.4, 7.2)**	**2.8 (1.4, 5.6)**	0.8 (0.2, 3.4)
Current sleep troubles	**1.8 (1.2, 2.8)**	**5.0 (3.4, 7.4)**	0.8 (0.5, 1.3)
Currently in poor physical health	**1.7 (1.2, 2.5)**	n/a	0.8 (0.5, 1.2)
			
*Previous psychiatric history*			
Adolescent mental disorder	1.2 (0.7, 2.4)	1.4 (0.7, 2.7)	1.6 (0.9, 3.0)
Treatment for psychiatric illness ages 16 to 36	**2.8 (1.8, 4.4)**	**2.1 (1.3, 3.5)**	**3.5 (2.2, 5.3)**
Self-reported history of nervous trouble	**7.3 (4.8, 11.0)**	**6.5 (4.3, 9.9)**	n/a
Previous psychotropic medication use	**7.7 (4.5, 13.4)**	**5.5 (3.5, 8.6)**	**6.4 (4.4, 9.4)**

Odds ratios **in bold** are significant at *p* < 0.05.

a Odds ratios for all variables, apart from mental disorder, are adjusted for severity of symptoms of mental disorder.

**Table 2:** Non-clinical predictors of those treated with psychotropic medications during an episode of mental disorder, adjusted for severity of mental disorder and previous psychotropic medication use

	Odd ratios for treatment (95% CI)^a^		
	1982	1989	1999
	Age 36	Age 43	Age 53
	*n* = 3248	*n* = 3197	*n* = 2928
*Personality*			
High neuroticism	**0.6 (0.4, 0.9)**	1.1 (0.7, 1.6)	**1.5 (1.1, 2.2)**
High extraversion	0.9 (0.6, 1.4)	0.7 (0.5, 1.0)	1.0 (0.7, 1.4)
			
*Social and related factors*			
Female	1.2 (0.8, 1.7)	**1.5 (1.1, 2.2)**	**1.4 (1.0, 1.9)**
Currently in manual social class	0.9 (0.6, 1.4)	1.0 (0.7, 1.5)	1.0 (0.7, 1.5)
Completed A-levels	0.8 (0.6, 1.2)	1.0 (0.7, 1.5)	0.8 (0.6, 1.2)
Divorced, separated, or widowed	1.6 (0.9, 2.6)	1.0 (0.6, 1.6)	**1.5 (1.1, 2.3)**
Currently living alone	0.8 (0.3, 2.0)	1.2 (0.6, 2.2)	**2.6 (1.7, 3.9)**
Current unemployment	**1.9 (1.2, 3.2)**	1.3 (0.6, 2.9)	**2.9 (2.1, 4.1)**
More than 1 stressful life event in past year	1.0 (0.7, 1.4)	1.0 (0.7, 1.5)	1.1 (0.8, 1.5)
			
*Parental and childhood factors*			
Father was manual social class	1.0 (0.7, 1.5)	0.9 (0.6, 1.3)	1.0 (0.7, 1.4)
Parents had nervous trouble	0.8 (0.5, 1.2)	**1.5 (1.0, 2.3)**	**1.6 (1.1, 2.4)**
Low cognitive ability	1.0 (0.7, 1.5)	1.0 (0.7, 1.5)	1.1 (0.8, 1.5)

Odds ratios **in bold** are significant at *p* < 0.05.

a Odds ratios for all variables are adjusted for severity of symptoms of mental disorder.
